# A Low-Jitter Harmonic-Free All-Digital Delay-Locked Loop for Multi-Channel Vernier TDC

**DOI:** 10.3390/s22010284

**Published:** 2021-12-31

**Authors:** Jiyun Tong, Sha Wang, Shuang Zhang, Mengdi Zhang, Ye Zhao, Fazhan Zhao

**Affiliations:** 1 Institute of Microelectronics of the Chinese Academy of Sciences, Beijing 100029, China; tongjiyun@ime.ac.cn (J.T.); wangsha@ime.ac.cn (S.W.); zhangshuang2020@ime.ac.cn (S.Z.); zhangmengdi@ime.ac.cn (M.Z.); zhaofazhan@ime.ac.cn (F.Z.); 2 University of Chinese Academy of Sciences, Beijing 100049, China; 3 Key Laboratory of Science and Technology on Silicon Devices, Chinese Academy of Sciences, Beijing 100029, China; 4 School of Information Science and Technology, North China University of Technology, Beijing 100144, China

**Keywords:** All-Digital Delay-Locked Loop (ADDLL), harmonic lock, modified binary search, dithering phenomenon, low jitter, Vernier TDC

## Abstract

This paper presents a low jitter All-Digital Delay-Locked Loop (ADDLL) with fast lock time and process immunity. A coarse locking algorithm is proposed to prevent harmonic locking with just a small increase in hardware resources. In order to effectively solve the dithering phenomenon after locking, a replica delay line and a modified binary search algorithm with two modes were introduced in our ADDLL, which can significantly reduce the peak-to-peak jitter of the replica delay line. In addition, digital codes for a replica delay line can be conveniently applied to the delay line of multi-channel Vernier TDC while maintaining consistency between channels. The proposed ADDLL has been designed in 55 nm CMOS technology. In addition, the post-layout simulation results show that when operated at 1.2 V, the proposed ADDLL locks within 37 cycles and has a closed-loop characteristic, the peak-to-peak and root-mean-square jitter at 800 MHz are 6.5 ps and 1.18 ps, respectively. The active area is 0.024 mm^2^ and the power consumption at 800 MHz is 6.92 mW. In order to verify the performance of the proposed ADDLL, an architecture of dual ADDLL is applied to Vernier TDC to stabilize the Vernier delay lines against the process, voltage, and temperature (PVT) variations. With a 600 MHz operating frequency, the TDC achieves a 10.7 ps resolution, and the proposed ADDLL can keep the resolution stable even if PVT varies.

## 1. Introduction

Delay-Locked Loops (DLLs) with high-locking accuracy and process immunity are extensively used in high resolution and high precision Time-to-digital Converter (TDC) [1,2,3]. In high-precision scenarios, such as LIDAR and PET (Positron Emission Tomography), we need the TDC to have sufficient channels to collect large amounts of information quickly and achieve high resolution and large dynamic measurement ranges simultaneously. The Vernier TDC can meet the above requirements well; it can achieve ultra-high resolution. With the combination of coarse count and fine count, it can achieve a large measurement range. Importantly, the performance of the Vernier TDC relies on the difference between the fast delay line and slow delay line and the stability of delay lines against PVT variations. It reminds us to focus on the construction of the Vernier delay line.

Basically, the delay line in the Vernier TDC can be roughly divided into three categories. The first kind of delay line is composed of typical inverters without feedback adjustment circuits [4,5,6,7,8]. This kind of delay line has a simple structure, but cannot deal with PVT variations, so its robustness needs to be improved. The second kind of delay line consists of the VCO (Voltage-Controlled Oscillator) of the PLL (Phase-Locked Loop) [9,10]; this kind of structure needs two PLLs to provide two different frequencies for the delay lines. The power consumption and area will be large, and it is hard to transfer between different technologies. The last kind of delay line is provided by the delay line of DLL [11,12,13]. When compared with PLL, DLL can achieve fast lock-in without jitter accumulation and has a better performance when PVT varies.

**Figure 1:**
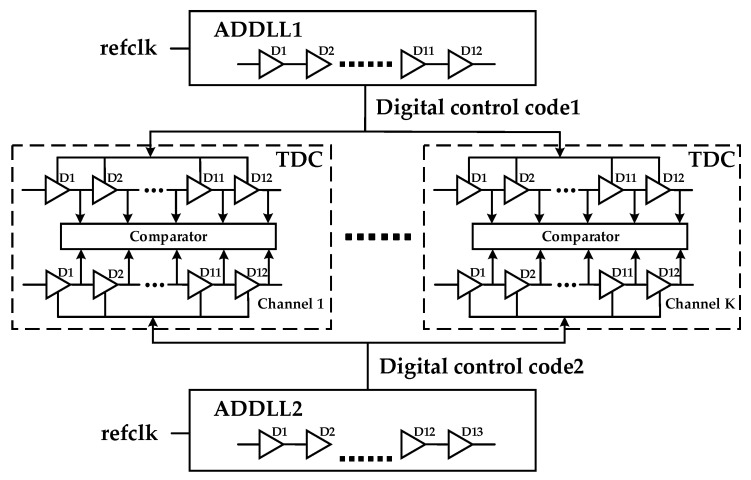
ADDLL applied to Multi-Channel Vernier TDC.

Generally, the goals of a DLL design we need to meet include a wide-operating frequency range, low jitter, fast locking, low power, and process immunity. Conventionally, analog DLLs can perform better in terms of jitter and skew, but they need a long locking time and large chip area, and they are sensitive to PVT variations, which makes them hard to migrate to advanced technologies. Compared with analog DLLs, all-digital DLL is a better choice because of its advantages of fast locking time, easy migration, high power efficiency, and effective tolerance of PVT variations. Furthermore, ADDLL can be better applied to multi-channel TDC because it only requires digital codes rather than analog voltage signals to control the delay line, which allows achieving better consistency between TDC channels. Based on the above merits, when we tried to apply DLL to Multi-channel Vernier TDC, ADDLL is preferred. The diagram of applying ADDLL to Multi-channel Vernier TDC is shown in Figure 1.

Usually, conventional ADDLL adopts a counter to adjust the delay line [14]. To take into account the wide frequency range together with the small delay resolution, the locking time of counter-based DLL will increase exponentially as the control bits increase. Therefore, a successive approximation register-controlled (SAR) circuit is employed to reduce the locking time [15], but as it is affected by its open-loop characteristic, additional calibration circuits should be taken into consideration to track the PVT variations, which not only increase the complexity of the whole DLL but also raise the power consumption. Then a variable successive approximation register-controlled (VSAR) algorithm is adopted to keep tracking the PVT variations after first locking [16,17]. The method transforms the VSAR circuits into a counter after the first locking, the counter adjusts the delay according to the changes in the external environment. On account of the finite step size of adjustment, the delay of the digitally controlled delay line (DCDL) may not be exactly equal to the clock period, even if the DLL is considered locked. So, the digital codes for a counter-based DLL will change back and forth around the edge of the reference clock which we called the dithering phenomenon [18]. The dithering phenomenon increases both the total power dissipation and output uncertainty. In order to solve the dithering phenomenon, a tri-state digital phase detector (TSDPD) which has an additional Hold region is used in [16,19]. Once the comparison result between the two signals in TSDPD falls into the HOLD region, the circuit is locked, and the dithering phenomenon is suppressed. However, we need to accurately control the range of the HOLD region which has to be slightly larger than the delay step. The drawback of this method is that the range of the HOLD region will change due to PVT variations, and it is hard to apply TSDPD to other DLLs which differs in delay step. Moreover, because the relationship between the initial delay time and the reference clock is unknown, harmonic locking issues may occur in the wide-operating frequency range DLL, which means the delay provided by DLL is a 720° phase or more instead of 360°, resulting in false locking. The widely used SAR DLL has the problem of harmonic locking. To deal with the harmonic locking issue, a SAR DLL is presented in [20] with a resettable linear lattice delay line (LDL). It has to work with the proposed LDL which limits its scope of application. A starting-bit prediction (SBP) algorithm is presented in [21] which can avoid harmonic locking issues effectively, but the design of the associated circuit is complex and resource-consuming. Furthermore, the maximum operating frequency under the SBP algorithm is limited by the intrinsic delay of the delay line, T_intrinsic_ ≤ T_clk_/2.

A low jitter, fast locking, closed-loop ADDLL which can eliminate harmonic locking issues and the dithering phenomenon is proposed in this work. It can provide a process insensitive delay line with high accuracy for multi-channel Vernier TDC. In the proposed ADDLL, a coarse locking method is proposed to solve the harmonic locking issue with just a few transistors increased. In order to reach our goal of dispelling the dithering phenomenon while keeping track of the PVT variations of the external environment, a replica delay line and a modified binary search algorithm were introduced. After the first locking of DLL, the target digital control code is applied to the replica delay line, while the main delay line still searches within a small range around the last target digital code. Unless the external operating condition changed, the output of the replica delay line will remain constant, in which situation the dithering phenomenon is suppressed.

The organization of this paper is as follows. Section 2 presents the architecture and circuit implementation details. The results and discussion are shown in Section 3. Finally, a brief conclusion has been presented in Section 4.

## 2. Proposed ADDLL Structure

**Figure 2:**
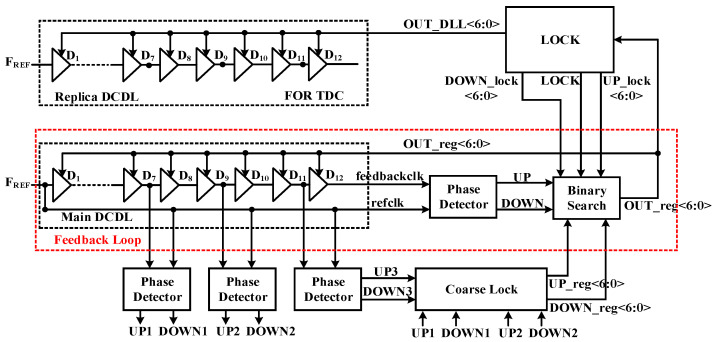
The architecture of the proposed ADDLL.

Figure 2 shows the architecture of the whole ADDLL, which comprises five major parts: digitally-controlled delay line (DCDL), a phase detector (PD), binary search module, coarse lock module, and lock module. Among them, the main DCDL, PD, and binary search modules form a closed feedback loop to track the PVT variations. Notice that the entire DLL includes two delay lines: the main delay line and the replica delay line. The overall operating mechanism is that the main delay line is constantly searching to track delay variations caused by the external environment, while the replica delay line is applied to TDC maintain in a stable state. The digital code OUT_reg<6:0> is used to control the delay of the main delay line, and OUT_DLL<6:0> is used to control the delay of the replica delay line.

**Figure 3:**
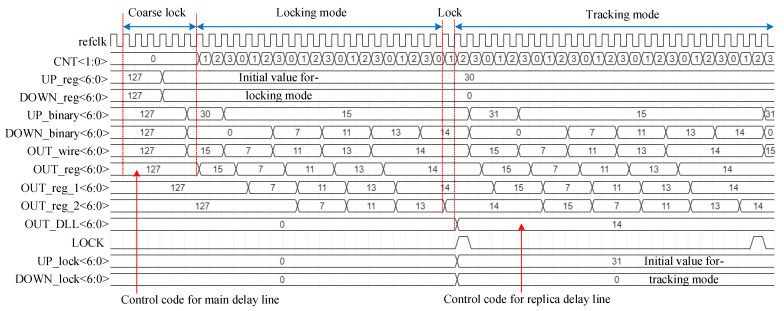
The timing diagram of the proposed ADDLL.

The timing diagram of the proposed ADDLL is shown in Figure 3. Different from the traditional scheme which is to divide delay units into the coarse delay unit and fine delay unit, the proposed ADDLL separates the locking procedure into coarse searching and fine searching, while the delay resolution of delay elements stays in its uniformity, which can both save area and power. After the DLL is powered on, first we use the coarse lock module to acquire a proper digital code for the main delay line according to the output of three PDs, which can help us to avoid harmonic locking. Then work with modified binary search algorithm continually until DLL is locked. After the first locking of DLL, the feedback loop keeps on searching around a specific range in response to PVT changes, while the delay time of the replica delay line remains stable. The modified binary search algorithm includes two modes: locking mode and tracking mode. The locking mode is used to obtain the first lock state, while the tracking mode is applied to track PVT variations after the first locking, and details will be explained later.

**Figure 4:**
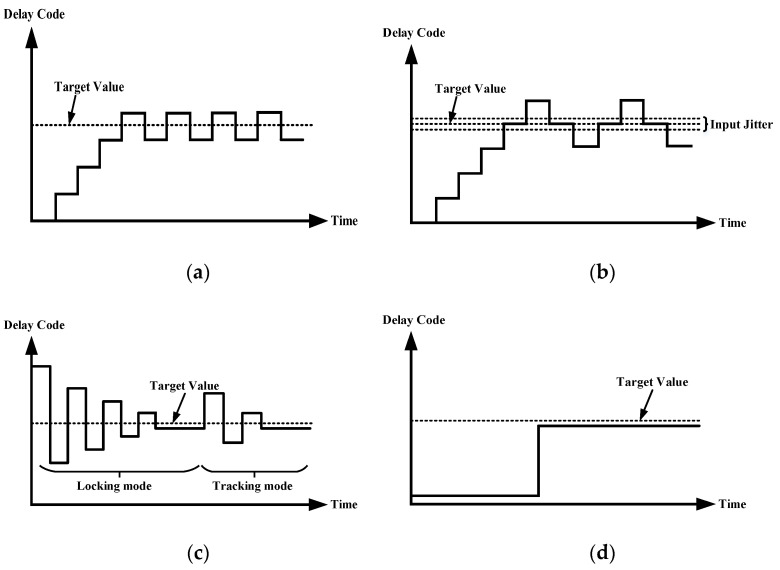
(**a**) Locking process of the counter-based DLL; (**b**) Worst case on counter-based DLL with a small input clock jitter; (**c**) Locking process of the main DCDL; (**d**) Control code of the replica DCDL.

The proposed modified binary search can suppress the dithering phenomenon effectively, as shown in Figure 4 A comparison has been made on the dithering phenomenon between counter-based DLL and proposed DLL. Figure 4a shows the dithering process of the counter-based DLL and its target delay of the DCDL, while Figure 4b depicts the worst case with a small jitter in the clock source considered. As shown in Figure 4c,d, after the first locking of DLL, the main DCDL works in tracking mode to track PVT variations, while the replica DCDL remains stable which means the dithering phenomenon is eliminated. This will significantly reduce the peak-to-peak jitter.

### 2.1. Phase Detector

**Figure 5:**
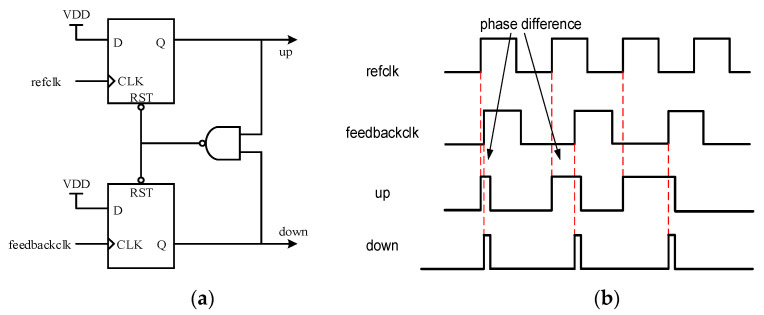
Traditional phase detector: (**a**) Architecture; (**b**) Timing waveform.

Respectively, Figure 5a,b shows the architecture and the timing waveform of a traditional phase detector, which consists of flip-flops and logic gates. Two main drawbacks exist in a traditional PD which make it hard to fit the demand of digital DLL. First, the pulse width of the output signal is proportional to the phase difference between the reference clock and feedback clock, resulting in ultra-narrow pulses when the phase difference approaches zero. Under this condition, the pulse width will become too narrow to be sampled by subsequent registers. Second, even if we can widen the pulse width of two output signals, which are named UP and DOWN, by using combinational logic, the pulse width difference between two output signals is still very small, which means the following sampling circuits cannot distinguish between them.

**Figure 6:**
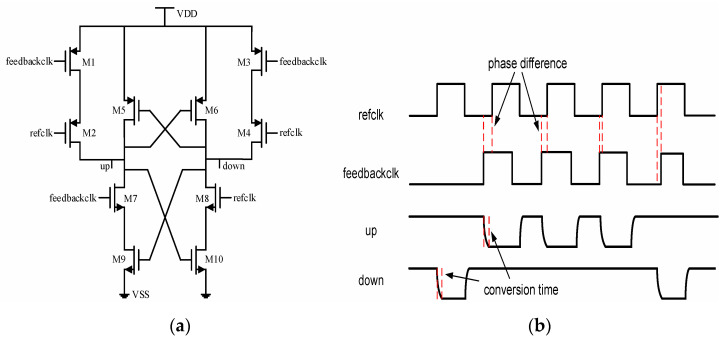
Phase detector used in proposed ADDLL: (**a**) Architecture; (**b**) Timing waveform.

For the purpose of avoiding the above problems of the traditional structure, a phase detector with a completely symmetrical structure is used [22], as shown in Figure 6a. It can function well in [−π, π] with no dead zone and the effective electrical level of the output signal is low-level. In the beginning, refclk and feedbackclk are both considered low-level. At this time, transistors M1–M4 are turned on, M7, M8 are turned off, nodes UP and DOWN are charged to a high-level, and then M5, M6 are turned off, and M9, M10 are turned on. If refclk turns to high before feedbackclk, M2 and M4 are turned off to stop charging, and M8 is turned on. Because M10 has been turned on in advance, the node DOWN will quickly discharge to a low level along the path of M8 and M10. After node DOWN becomes low, turn off M9 and turn on M5. Node UP is stuck at high-level, and our effective low-level signal DOWN is output. After refclk and feedbackclk both become low, the signals UP and DOWN both change back to the initial high-level, which is the initialization for the next comparison. Similarly, if feedbackclk turns to high before refclk, the circuit will output an effective low-level signal UP. As we can see in Figure 6b, the pulse width of the output signals is stretched to at least half of the reference clock cycle regardless of how small the phase difference is. It is worth noting that the conversion time of the signal is not negligible. If the subsequent circuit sampling UP and DOWN signals activate the rising edge of the reference clock named refclk, an incorrect value may be sampled. In response to this situation, a buffer is inserted in the clock path to ensure that the register can sample the expected value.

### 2.2. Delay Element

**Figure 7:**
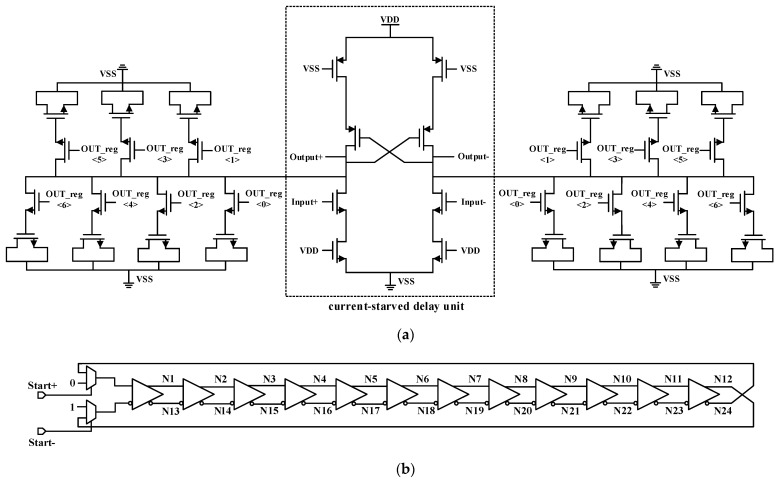
(**a**) A digitally controlled symmetrical current-starved delay unit; (**b**) Diagram of the delay line in Vernier TDC.

To employ our delay line in multi-channel TDC, a digitally controlled delay element with controllable capacitance is proposed and the structure is shown in Figure 7a. The main part is a symmetrical current-starved delay unit which is able to keep the delay resolution of rising edge and falling edge as consistent as possible. In order to achieve high resolution and wide measurement range at the same time, the delay line of the Vernier TDC is generally connected as a ring [23,24]. Catering for the requirement mentioned, the input and output ports of our delay unit are differential, which can be connected into a circular delay line, as shown in Figure 7b. It is obvious that only 12 delay units can generate 24 phases, which can significantly reduce the area of multi-channel TDC. The digital signal OUT_reg<6:0> is used as the control bit for the delay element to adjust the state of switching transistors. The state of the switching transistor can affect the size of load capacitance, thereby affecting the delay time of the delay unit. In the proposed delay element, the delay resolution of a single delay unit is 0.71 ps in the 25 °C TT process, and the delay range of a delay unit is 84.3 ps to 174.5 ps. The entire delay line contains 12 delay units, hence the delay resolution of the entire delay line is 8.52 ps.

### 2.3. Coarse Lock

**Figure 8:**
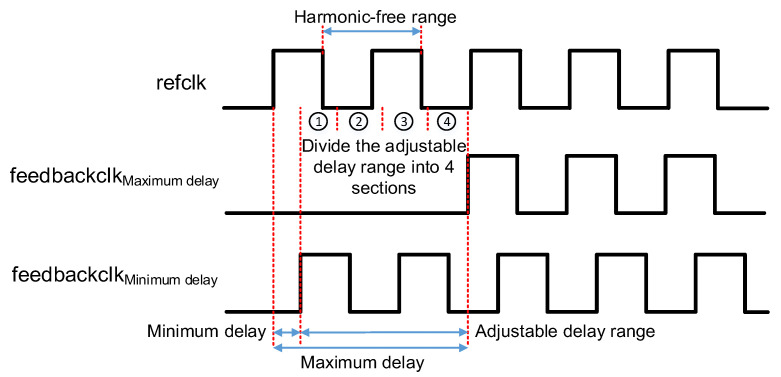
Diagram of the segmented method.

**Figure 9:**
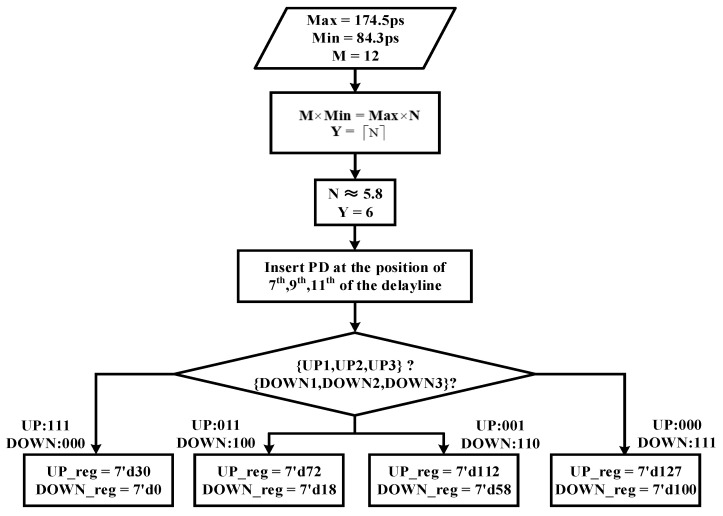
The process of coarse locking.

A DLL that can operate in a wide frequency range may have the problem of harmonic locking. This requires us to perform a coarse locking in advance to adjust the delay of the delay line near the target value. Therefore, three PDs are added outside the DLL feedback loop to find out the state of the phase difference with only 30 transistors increased, as shown in Figure 2. By inserting these three PDs in proper positions of the delay line, the adjustable delay range is divided into 4 sections. After the entire circuit is reset, the delay time of the delay unit is set to its maximum, which means the value of the 7-bit control code is 7’b1111111. Then the position of the minimum lockable clock period in the delay line can be calculated by the formula shown below:

(1) $$\begin{array}{c} M\times \mathrm{Min}=\mathrm{Max}\times N\\ Y=\left\lceil N\right\rceil \end{array}$$

where ⌈⌉ denotes rounding up the rational number to an integer, thus Y means the first position where we can insert PD. Min and Max denote the minimum delay and maximum delay of a single delay unit, respectively. M represents the number of delay units in the entire delay line and N represents the position of the minimum lockable clock period in the delay line which has a maximum delay. Position N to position M represents the adjustable range of our delay, the same as the 7-bit control code varies from 7′d0 to 7′d127. After dividing the adjustable delay range into four segments as evenly as possible, and then according to the output of the three PDs, we can adjust the delay to one of them, corresponding diagram, as shown in Figure 8. Then we start the binary search in the selected segment. Take the proposed ADDLL as an example, the detailed coarse locking process is as shown in Figure 9, and the delay value used is obtained in the 25 °C TT process.

**Figure 10:**
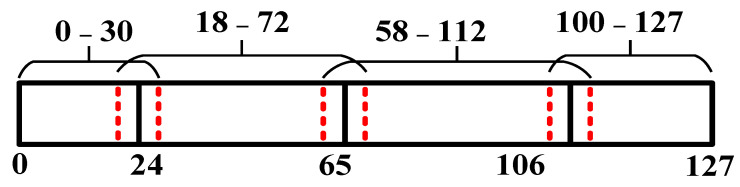
Segmentation of adjustable delay range.

According to the data above, PDs are inserted at the 7th, 9th, and 11th positions of the delay line, and our adjustable 7-bit delay range is divided into four segments: 0–24, 24–65, 65–106, 106–127. If the target control code happens to be at the edge of a segment, a false coarse locking may occur due to the influence of jitter and net delay. To avoid the false coarse locking mentioned before, the edge of each segment has been extended without increasing the locking period, as shown in Figure 10.

**Figure 11:**
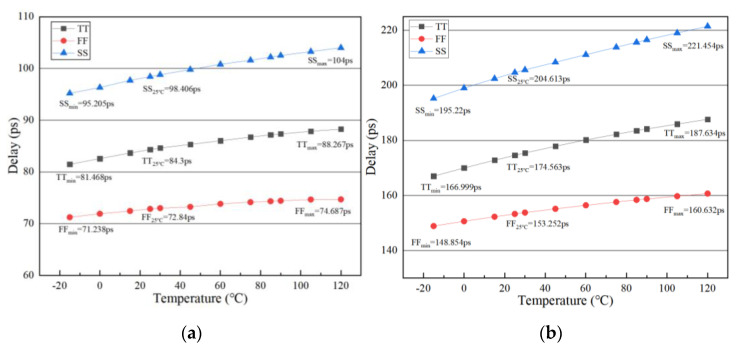
The delay variations of a single delay unit (**a**) OUT_DLL<6:0> = 7′b0000000; (**b**) OUT_DLL<6:0> = 7′b1111111.

Taking temperature variations and different processes into consideration, the segmentation method of coarse lock can still be applied. When the control code OUT_DLL<6:0> is 7’b0000000 and 7’b1111111, respectively, the delay variations of a single delay unit are recorded with post-layout simulation, as shown in Figure 11. We supposed that temperature and process will not change at the same time. After the calculation of all cases, we found that the maximum value of N was 5.854 which was obtained in case TT_min_, and the corresponding segmentation result was: 0–23, 23–65, 65–107, 107–127. The minimum value of N was 5.58 which was obtained in case FF_max_, the corresponding segmentation result was: 0–28, 28–68, 68–108, 108–127. It is obvious that both of the worst-case scenarios were covered by our extended segmentation which was: 0–30, 18–72, 58–112, 100–127. This means that the proposed coarse locking method is correct at different processes and temperatures.

### 2.4. Modified Binary Search

**Figure 12:**
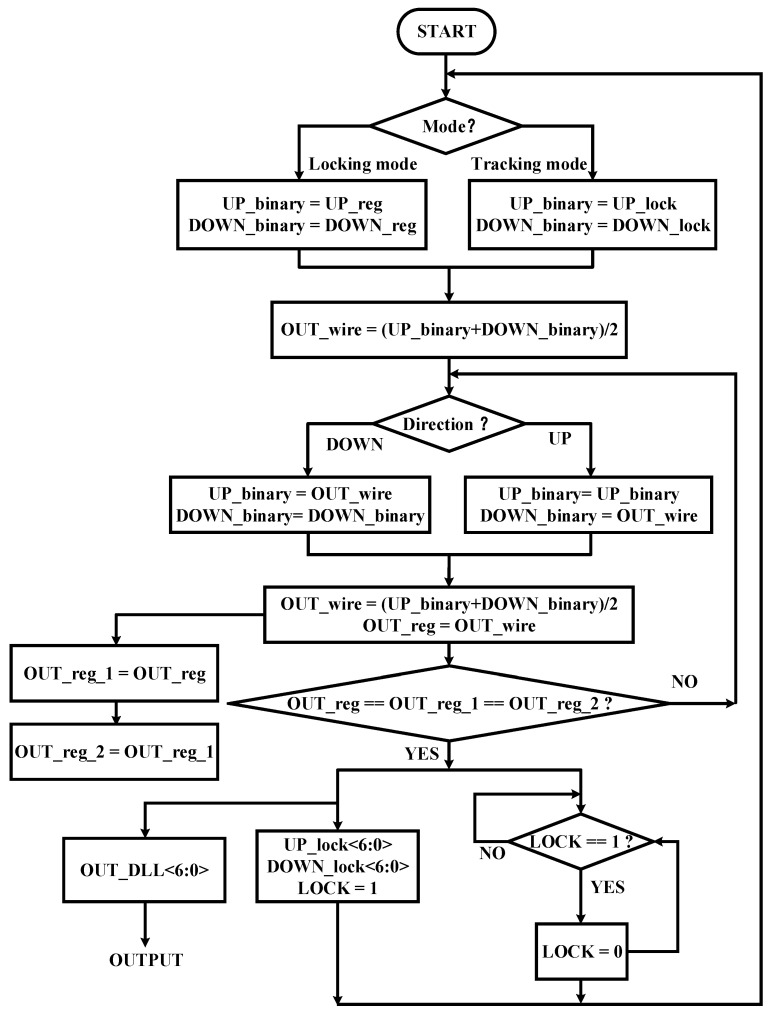
The flow chart of the proposed modified binary search algorithm.

After coarse locking, we need to find the exact control code for the delay line. A conventional binary search can quickly converge to the target value, but it has an open-loop characteristic which makes it unable to adjust the delay according to the changes in PVT. The flow chart of the proposed modified binary search algorithm is shown in Figure 12. It works with two modes: locking mode and tracking mode. A different initial value is assigned to the binary search module to start the searching process in a different mode. In locking mode, first, the segment of target delay is selected by coarse locking, after that, UP_reg<6:0> and DOWN_reg<6:0> of the chosen segment to the binary search module as the initial value is assigned. Then judge whether it has been locked through the lock module; once locked, it will output a high-level signal named LOCK which is maintained for one clock cycle. The signal LOCK is used to determine the working mode. If LOCK goes too high, the circuit goes into tracking mode. Then by assigning UP_lock<6:0> and DOWN_lock<6:0> to the binary search module as the initial value, UP_lock<6:0> and DOWN_lock<6:0> are output by the lock module. LOCK = 1 means that the search of the main delay line has reached a temporarily stable state, and at this time OUT_reg<6:0> is assigned to OUT_DLL<6:0>. Then use OUT_DLL<6:0> to control the delay of the replica delay line. After that, the main delay line performs a small range search near the output control code OUT_DLL<6:0>. The search range is between UP_lock<6:0> and DOWN_lock<6:0>. If the external environment does not change, the main delay line will be locked at the previous value again and output the same OUT_DLL<6:0>, otherwise, it will output a new OUT_DLL<6:0> according to the external changes. In tracking mode, the main delay line continues to perform a binary search in a small range according to the values of UP_lock<6:0> and DOWN_lock<6:0>; therefore, a closed loop is formed to track the variations of PVT. Moreover, during the tracking mode, the delay of the replica delay line will remain stable unless the external environment change, and we take the control code of the replica delay line as the final output of the DLL which means it can be applied to multiple delay lines without dithering.

**Figure 13:**
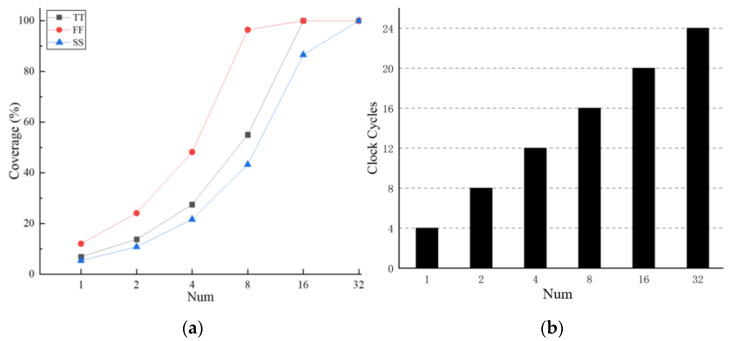
(**a**) Coverage of the delay change; (**b**) Clock cycles required for searching in tracking mode.

When it comes to the searching range of the main delay line after the first locking, UP_lock<6:0> and DOWN_lock<6:0> will be the initial value of the following searching process during tracking mode. The values of UP_lock<6:0> and DOWN_lock<6:0> are obtained by adding or subtracting a certain number named Num to OUT_DLL<6:0>. The selection of Num needs to take into account the clock cycles required for locking and the delay variations caused by the change in temperature. The delay change in a single delay unit caused by temperature variations is shown in Figure 11. In addition, the maximum delay change caused by the temperature variation was 26.234 ps, 20.635 ps, and 11.778 ps at three different process corners SS, TT, and FF. When adding or subtracting different values of Num, we analyzed the Coverage of the delay change and the clock cycles required for searching, as shown in Figure 13a,b, respectively.

As shown in Figure 11, there is a certain range of the delay variation caused by temperature. UP_lock<6:0> minus DOWN_lock<6:0> can get the search range of the tracking mode. The Coverage means the coverage percentage of the search range in tracking mode to the range of delay variations caused by temperature. The Coverage is calculated by the formula shown below:

(2) $$\mathrm{Coverage}=\frac{\mathrm{searching}\;\mathrm{range}\;\mathrm{in}\;\mathrm{tracking}\;\mathrm{mode}}{\mathrm{delay}\;\mathrm{variations}}=\frac{\mathrm{Num}\times 0.71\times 2}{{\mathrm{delay}}_{\max}-{\mathrm{delay}}_{\min}}\times 100\%$$

where 0.71 is the delay resolution of a single delay unit. The delay_max_ and delay_min_ in different process corners are shown in Figure 11. For example, under SS process corner, OUT_DLL = 7′b1111111, take the value of addition or subtraction as 16:

(3) $$\mathrm{Coverage}=\frac{\mathrm{Num}\times 0.71\times 2}{{\mathrm{delay}}_{\max}-{\mathrm{delay}}_{\min}}\times 100\%=\frac{16\times 0.71\times 2}{221.454-195.22}\times 100\%\approx 86.6\%$$

It can be seen from Figure 13a,b that the Coverage rate and the clock cycles required for locking both increase with the increase in the Num. When the value of Num is 16, almost 100% Coverage can be achieved under 3 different process corners, and the clock cycle required for locking is less than those numbers which can also achieve 100% Coverage because we checked whether “OUT_reg<6:0> = OUT_reg_1<6:0> = OUT_reg_2<6:0>” was satisfied to judge whether the DLL was temporarily locked. If we add and subtract the same value of Num to get the value of UP_lock<6:0> and DOWN_lock<6:0>, the output digital code OUT_reg<6:0> will be the same as the last one. Then the LOCK signal will go wrong which will influence the tracking process. Therefore, we added 17 and subtracted 15 to OUT_DLL<6:0> to get the value of UP_lock<6:0> and DOWN_lock<6:0>, respectively.

### 2.5. Lock

**Figure 14:**
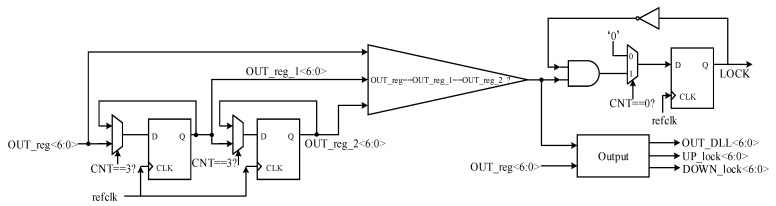
Circuit diagram of the lock module.

The function of the lock module is mainly to switch the mode of the circuit, output the corresponding LOCK signal, and output digital control code OUT_DLL<6:0> which is applied to the replica delay line. The circuit diagram is shown in Figure 14. Every time it takes 4 clock cycles to output the digital control code OUT_reg<6:0> is applied to the main delay line, hence a counter named CNT that counts from 0 to 3 with a period of 4 is employed. Sampling OUT_reg<6:0> when CNT equals to 3 to get the value of OUT_reg_1<6:0> and OUT_reg_2<6:0>. If the above three signals are equivalent, the LOCK signal will be pulled up for one clock cycle when CNT is equal to 0 in the next clock cycle. At the same time, output the corresponding 7bit control code OUT_DLL<6:0> to adjust the delay of the replica delay line, and output UP_lock<6:0> and DOWN_lock<6:0> as the initial searching value for tracking mode.

## 3. Results and Discussion

**Figure 15:**
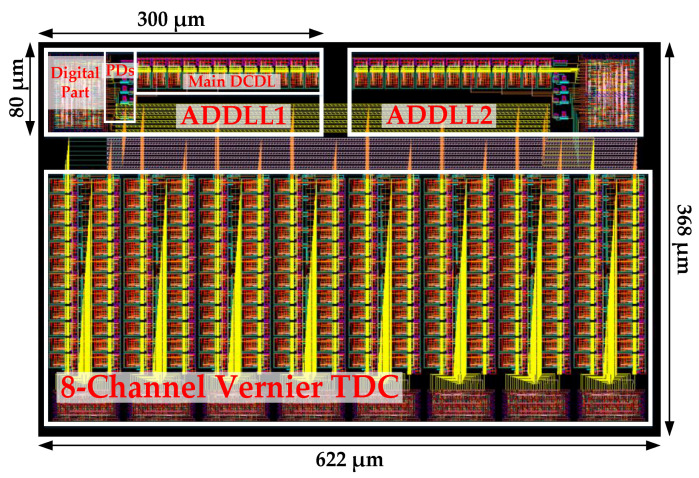
Layout of the proposed ADDLL and Vernier TDC of 8 channels.

The proposed ADDLL was designed in 55 nm CMOS technology. The power supply voltage was 1.2 V. Figure 15 shows the layout of the proposed ADDLL and eight-channel Vernier TDC, where the active area of an ADDLL was about 0.024 mm^2^. Multi-channel TDC allowed us to collect more information at the same time, while Vernier architecture ensured high precision and high resolution for TDC. When the proposed ADDLL operated at 800 MHz, the power consumption was about 6.92 mW. The required reference clock cycles for locking were 29 to 37.

**Figure 16:**
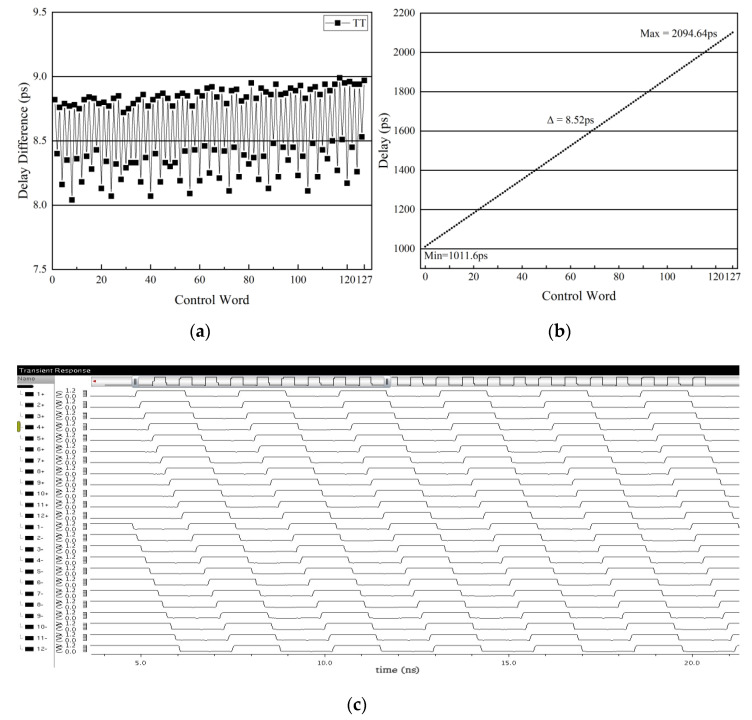
(**a**) Delay resolution at the process corner TT in 25 °C (**b**) Relationship between the delay time and the 7 bit control code; (**c**) The 24 phases provided by the replica delay line to the Vernier TDC.

The post-layout simulation result showed the variation of the delay resolution in the 25 °C, TT process corner, as depicted in Figure 16a. It can be seen that when the 7-bit control code varied from 0 to 127, the delay resolution was always between 8 ps and 9 ps. The maximum value was 8.99 ps, and the minimum value was 8.04 ps. The delay resolution of the entire delay line was small and had good uniformity. Figure 16b describes the relationship between the delay time of the whole delay line and the 7-bit control code. The delay range of the entire delay line was from 1011.6 ps to 2094.64 ps, and the average step size was about 8.52 ps. It means that in the 25 °C, TT process corner, the operating frequency range was 480 MHz to 980 MHz. Taking into account variations in temperature and process, the operating frequency range of the proposed ADDLL was 560 MHz to 800 MHz. The 24 phases provided by the replica delay line to the Vernier TDC are shown in Figure 16c, and the simulation result was obtained at 600 MHz and showed good linearity.

**Figure 17:**
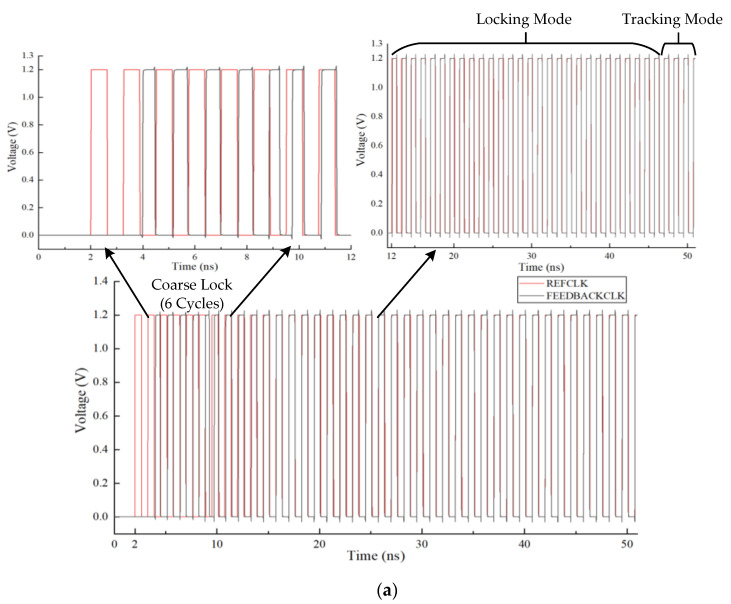

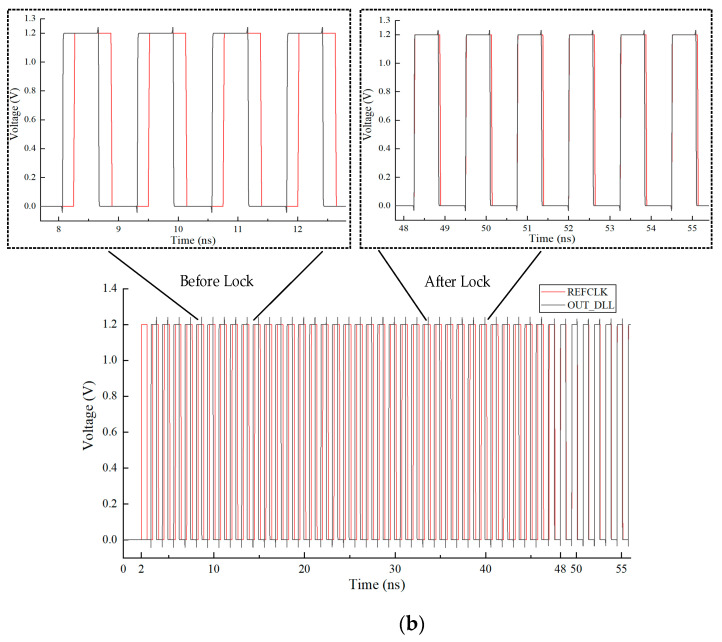
(**a**) Locking process of the main delay line at 800 MHz; (**b**) State of the replica delay line at 800 MHz.

Figure 17a shows the locking process of the main delay line at 800 MHz, while Figure 17b shows the state of the replica delay line, and the corresponding cycles for locking were 37. After power-on, because the delay line had not output a valid clock signal in the first clock cycle, the comparison result of the phase detector was not sampled, and then the normal operation of the circuit started from the second clock cycle. First, it performed the coarse lock to roughly estimate the delay time, thus adjusting the delay to the vicinity of the reference clock. It not only reduced the range that needed to be searched in the subsequent lock process but also avoided the occurrence of harmonic lock issues. The coarse lock consumed six clock cycles. Then it entered the binary search process with locking mode. Since each search process took four reference clock cycles, the 7-bit output code OUT_reg<6:0>, which was applied to the main delay line, changed at every four reference clock cycles. For different reference clocks, the locking situation of coarse lock may be diverse. Therefore, there a slight difference in searching range in the subsequent binary search might occur. For different searching ranges, five to seven search operations might be required, that is, 20 to 28 clock cycles, to reach the locked state. Then, a clock cycle might be required to determine whether the locked state has been reached. Finally, it took one clock cycle for the replica delay line to keep stable with a new control code OUT_DLL<6:0>.

**Figure 18:**
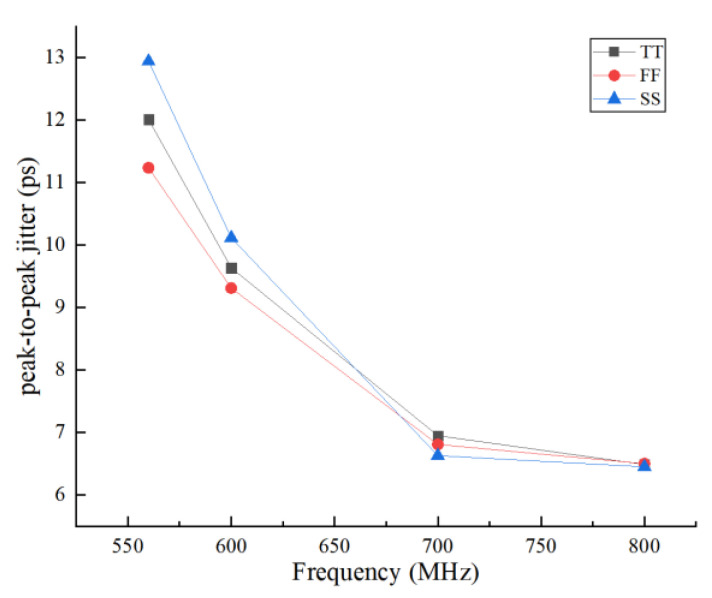
The variation of peak-to-peak jitter with respect to a different process.

Figure 18 shows the variation of peak-to-peak jitter with respect to different processes at several frequencies. We know from the results that the p-p jitter was smaller at a higher frequency, this is because the delay unit turns on more transistors at lower frequencies, making the delay more sensitive to changes in the process. The smaller the frequency, the larger the p-p jitter interval between different processes.

**Figure 19:**
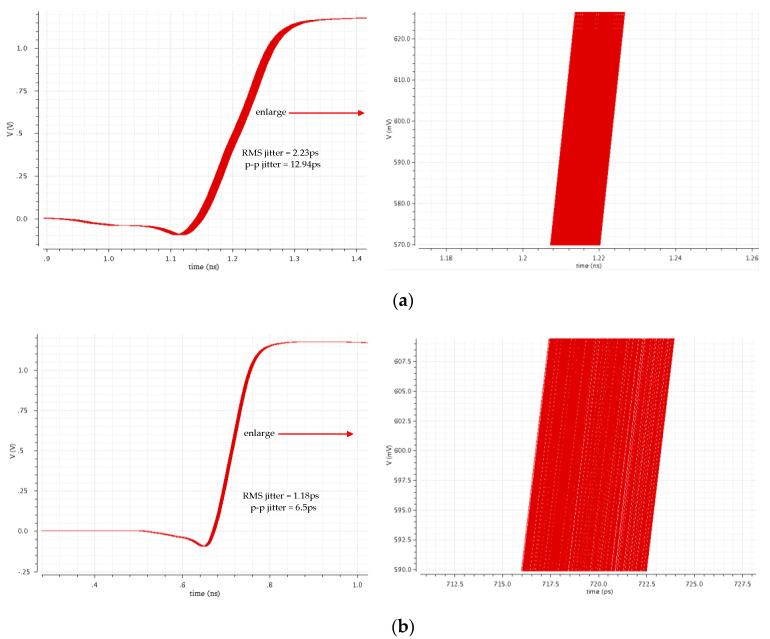
Simulated RMS jitter and p-p jitter of the replica delay line (**a**) 560 MHz; (**b**) 800 MHz.

Figure 19 shows the simulated jitter of the proposed ADDLL. It achieved a peak-to-peak (p-p) jitter of 12.94 ps and a root-mean-square (RMS) jitter of 2.23 ps at 560 MHz. When operated at 800 MHz, p-p jitter and RMS jitter were 6.5 ps and 1.18 ps, respectively. As mentioned, the low peak-to-peak jitter is attributed to the use of modified binary search and replica delay line, which eliminate the dithering phenomenon of the digital control code DLL_OUT<6:0>.

A performance comparison between the proposed All-Digital Delay-Locked Loop and the previous ADDLL was made, as presented in Table 1. Among the ADDLLs, the proposed ADDLL performed without harmonic locking issues and achieved very low jitter. Especially in jitter performance, the proposed ADDLL achieved the lowest jitter when compared with others in Table 1. This is attributed to the elimination of the dithering phenomenon by the proposed modified binary search and the replica delay line. Furthermore, the digital control code without dithering phenomenon can be applied to multiple delay lines, which makes our proposed ADDLL very suitable for multi-channel Vernier TDC. The proposed ADDLL also achieved high performance in terms of lock-in time, power dissipation, active area, and process immunity.

**Figure 20:**
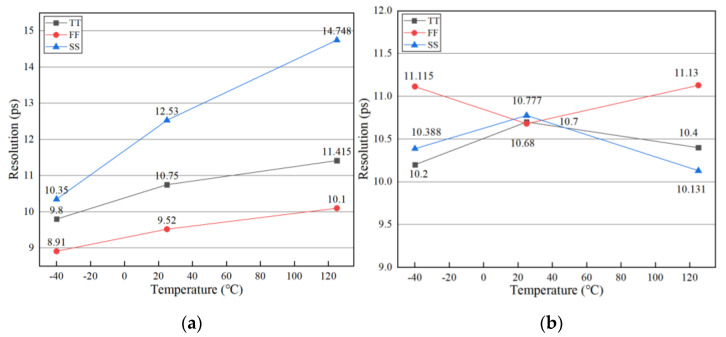
Resolution in Vernier TDC (**a**) Delay line without DLL; (**b**) Delay line with proposed ADDLL.

At the operating frequency of 600 MHz, we applied the proposed ADDLL to the Vernier TDC. When PVT varied, the resolution of TDC was obtained through simulation. We then compared the TDC resolution between the delay line provided by ADDLL and the same delay line without a feedback loop. Simulation results are shown in Figure 20.

From the simulation results in Figure 20, it is obvious that the stability of TDC resolution was better when we used the delay line provided by the proposed ADDLL than when using the ordinary delay line. Especially under the condition of 25 °C and the TT process, the resolution of the Vernier TDC with the proposed ADDLL was very stable. This shows the good performance of ADDLL even if the PVT varies.

## 4. Conclusions

A low jitter ADDLL with fast lock time and process immunity was presented in this paper. The ADDLL adopted a coarse lock method to avoid harmonic locking issues with few resources consumed. Meanwhile, by combining the modified binary search method with the replica delay line, the ADDLL tracked the PVT variations and eliminated the dithering phenomenon. The simulation result showed that the proposed ADDLL, which was designed in 55-nm 1.2-V CMOS technology, could operate at a maximum frequency of 800 MHz with a power consumption of 6.92 mW and 6.5 ps peak-to-peak jitter. Moreover, the ADDLL we designed can control multiple delay lines at the same time through digital control codes, which makes it suitable for multi-channel Vernier TDC. The delay lines provided by ADDLL for the Vernier TDC have high uniformity and low jitter. When the proposed ADDLL was applied to the Vernier TDC, the ADDLL ensured high time resolution even if the PVT varies.

## Figures and Tables

**Table 1:** All-Digital Delay-Locked Loop performance summary and comparison.

Reference	[20] Fab.	[25] Fab.	[26] Fab.	[27] Sim.	[28] Sim.	This Work Sim.
Process	180 nm	45 nm	65 nm	180 nm	65 nm	55 nm
Supply voltage (V)	1.8	1.1	1	1.8	1	1.2
Operating frequency range (MHz)	60–1200	400–800	700–2000	20–625	2000–3000	560–800
Locking time (cycles)	33	——	40	27–75	15	29–37
Jitter_p-p_ (ps)	17@600 MHz 12.8@1.2 GHz	12.9@800 MHz	22@2 GHz	8.6@625 MHz	17.46@2.4 GHz	6.5@800 MHz
Jitter_RMS_ (ps)	2.09@600 MHz 1.63@1.2 GHz	1.95@800 MHz	2.859@2 GHz	1.6@625 MHz	2.58@2.4 GHz	1.18@800 MHz
Power (mW)	16.2@1.2 GHz	1.32@800 MHz	3.31@1 GHz	7.85@625 MHz	3.3@2.4 GHz	6.92@800 MHz
Active area (mm^2^)	0.09	0.0034	0.019	——	0.043	0.024

## Data Availability

The data presented in this study are available on request from the corresponding author. The data are not publicly available due to privacy.

## References

[1] Hejazi A., Oh S., Rehman M.R.U., Rad R.E., Kim S., Lee J., Pu Y., Hwang K.C., Yang Y., Lee K.-Y. (2020). A Low-Power Multichannel Time-to-Digital Converter Using All-Digital Nested Delay-Locked Loops With 50-ps Resolution and High Throughput for LiDAR Sensors. IEEE Trans. Instrum. Meas..

[2] Jansson J.-P., Koskinen V., Mantyniemi A., Kostamovaara J. (2012). A Multichannel High-Precision CMOS Time-to-Digital Converter for Laser-Scanner-Based Perception Systems. IEEE Trans. Instrum. Meas..

[3] Marco Z., Paolo M., Salvatore L., Carlo S., Andrea L.L. (2010). Time-to-Digital Converter for Frequency Synthesis Based on a Digital Bang-Bang DLL. IEEE Trans. Circuits Syst. I.

[4] Omar H.H., Kareem R.R., Faisal A.H., Mohamed M.A. A 4-bit 2ps Resolution Time-to-Digital Converter Utilizing Multi-Path Delay Line for ADPLL. Proceedings of the 2019 31st International Conference on Microelectronics (ICM).

[5] Yu J.J., Fa F.D., Richard C.J. (2010). A 12-Bit Vernier Ring Time-to-Digital Converter in 0.13 μm CMOS Technology. IEEE J. Solid State Circuits.

[6] Mostafa R. Multi-step and high-resolution vernier-based TDC architecture. Proceedings of the 2017 29th International Conference on Microelectronics (ICM).

[7] Annagrebah A., Bechetoille E., Laktineh I.B., Chanal H. A Multi-Phase Time-to-Digital Converter Differential Vernier Ring Oscillator. Proceedings of the 2019 IEEE 10th Annual Ubiquitous Computing, Electronics & Mobile Communication Conference (UEMCON).

[8] Wu J., Deng W., Chen Z., Zheng W., Liu Y., Wang S. (2020). A 77-GHz Mixed-Mode FMCW Generator Based on a Vernier TDC with Dual Rising-Edge Fractional-Phase Detector. IEEE Trans. Circuits Syst. I.

[9] Chen P., Chen C., Zheng J.C., Shen Y. (2007). A PVT Insensitive Vernier-Based Time-to-Digital Converter with Extended Input Range and High Accuracy. IEEE Trans. Nucl. Sci..

[10] Kim M., Shin W.Y., Hong G.M., Park J., Chae J.H., Xing N. High-resolution and wide-dynamic range time-to-digital converter with a multi-phase cyclic Vernier delay line. Proceedings of the ESSCIRC (ESSCIRC).

[11] Bojan M., Simone T., Federica A.V., Alberto T., Franco Z. (2013). A High-Linearity, 17 ps Precision Time-to-Digital Converter Based on a Single-Stage Vernier Delay Loop Fine Interpolation. IEEE Trans. Circuits Syst. I.

[12] Noble N.-T., Alex T., Samuel P. A 15b, Sub-10ps resolution, low dead time, wide range two-stage TDC. Proceedings of the 2014 IEEE 57th International Midwest Symposium on Circuits and Systems (MWSCAS).

[13] Vincenzo S., Federica V., Enrico C., Alberto T. A novel sub-10 ps resolution TDC for CMOS SPAD array. Proceedings of the 25th IEEE International Conference on Electronics, Circuits and Systems (ICECS).

[14] Pasha M.T., Shah Y.A., Wikner J. A wide range all-digital delay locked loop for video applications. Proceedings of the 2015 European Conference on Circuit Theory and Design (ECCTD).

[15] Kuo K.-C., Li S.-H. A wide-range and harmonic-free SAR all-digital delay locked loop. Proceedings of the 2015 15th International Symposium on Communications and Information Technologies (ISCIT).

[16] Wang J.-S., Cheng C.-Y., Liu J.-C., Liu Y.-C., Wang Y.-M. (2010). A Duty-Cycle-Distortion-Tolerant Half-Delay-Line Low-Power Fast-Lock-in All-Digital Delay-Locked Loop. IEEE J. Solid State Circuits.

[17] Wang J.-S., Cheng C.-Y., Chou P.-Y., Yang T.-Y. (2015). A Wide-Range, Low-Power, All-Digital Delay-Locked Loop with Cyclic Half-Delay-Line Architecture. IEEE J. Solid State Circuits.

[18] Behzad M., Atila A. (2009). A Low-Power Digital DLL-Based Clock Generator in Open-Loop Mode. IEEE J. Solid State Circuits.

[19] Yang R.-J., Liu S.-I. (2007). A 2.5 GHz All-Digital Delay-Locked Loop in 0.13 μm CMOS Technology. IEEE J. Solid State Circuits.

[20] Yao C.-Y., Ho Y.-H., Chiu Y.-Y., Yang R.-J. (2015). Designing a SAR-Based All-Digital Delay-Locked Loop With Constant Acquisition Cycles Using a Resettable Delay Line. IEEE Trans. Very Large Scale Integr. (VLSI) Syst..

[21] Ho Y.-H., Yao C.-Y. (2016). A Fast-Acquisition All-Digital Delay-Locked Loop Using a Starting-Bit Prediction Algorithm for the Successive-Approximation Register. IEEE Trans. Very Large Scale Integr. (VLSI) Syst..

[22] Sobhan S., Farhad R., Mohammad G. (2019). Low-Power High-Frequency Phase Frequency Detector for Minimal Blind-Zone PhaseLocked Loops. Circuits Syst. Signal Process..

[23] Cheng Z., Deen M.J., Peng H. (2016). A Low-Power Gateable Vernier Ring Oscillator Time-to-Digital Converter for Biomedical Imaging Applications. IEEE Trans. Biomed. Circuits Syst..

[24] Zhang J., Zhou D.-M. (2018). An 8.5-ps Two-Stage Vernier Delay-Line Loop Shrinking Time-to-Digital Converter in 130-nm Flash FPGA. IEEE Trans. Instrum. Meas..

[25] Jung D.-H., Ryu K., Park J.-H., Jung S.-O. (2016). All-Digital 90° Phase-Shift DLL With Dithering Jitter Suppression Scheme. IEEE Trans. Very Large Scale Integr. (VLSI) Syst..

[26] Kim J., Han S.-W. (2018). A Fast-Locking All-Digital Multiplying DLL for Fractional-Ratio Dynamic Frequency Scaling. IEEE Trans. Circuits Syst. II.

[27] Kazeminia S., Abdollahi R., Hejazi A. (2018). A fast-locking low-jitter digitally-enhanced DLL dynamically controlled for loop-gain and stability. Analog. Integr. Circuits Signal Process..

[28] Park D., Choi S., Kim J. (2021). A Fast Lock All-Digital MDLL Using a Cyclic Vernier TDC for Burst-Mode Links. Electronics.

